# Orf virus DNA vaccines expressing ORFV 011 and ORFV 059 chimeric protein enhances immunogenicity

**DOI:** 10.1186/1743-422X-8-562

**Published:** 2011-12-29

**Authors:** Kui Zhao, Wenqi He, Wei Gao, Huijun Lu, Tiesuo Han, Jing Li, Ximu Zhang, Bingbing Zhang, Gaili Wang, Gaoli Su, Zhihui Zhao, Deguang Song, Feng Gao

**Affiliations:** 1 College of Animal Science and Veterinary Medicine, Jilin University, Changchun 130062, China; 2 Laboratory Animal Center, Jilin University, Changchun 130062, China; 3 Key Laboratory of Zoonosis, Ministry of Education, Institute of Zoonosis and Animal Research Center, Jilin University, Changchun 130062, China; 4 Laboratory Animal Center, Peking University, Beijing 100871, China

## Abstract

**Background:**

ORFV attenuated live vaccines have been the main prophylactic measure against contagious ecthyma in sheep and goats in the last decades, which play an important role in preventing the outbreak of the disease. However, the available vaccines do not induce lasting immunity in sheep and goats. On the other hand, variation in the terminal genome of Orf virus vaccine strains during cell culture adaptation may affect the efficacy of a vaccine. Currently, there are no more effective antiviral treatments available for contagious ecthyma.

**Results:**

We constructed three eukaryotic expression vectors pcDNA3.1-ORFV011, pcDNA3.1-ORFV059 and pcDNA3.1-ORFV011/ORFV059 and tested their immunogenicity in mouse model. High level expression of the recombinant proteins ORFV011, ORFV059 and ORFV011/ORFV059 was confirmed by western blotting analysis and indirect fluorescence antibody (IFA) tests. The ORFV-specific antibody titers and serum IgG1/IgG2a titers, the proliferation of lymphocytes and ORFV-specific cytokines (IL-2, IL-4, IL-6, IFN-γ, and TNF-α) were examined to evaluate the immune responses of the vaccinated mice. We found that mice inoculated with pcDNA3.1-ORFV 011/ORFV059 had significantly stronger immunological responses than those inoculated with pcDNA3.1-ORFV011, pcDNA3.1-ORFV059, or pcDNA3.1-ORFV011 plus pcDNA3.1-ORFV059. Compared to other vaccine plasmids immunized groups, pcDNA3.1-ORFV011/ORFV059 immunized group enhances immunogenicity.

**Conclusions:**

We concluded that DNA vaccine pcDNA3.1-ORFV011/ORFV059 expressing ORFV011 and ORFV059 chemeric-proteins can significantly improve the potency of DNA vaccination and could be served as more effective and safe approach for new vaccines against ORFV.

## Background

Orf virus (ORFV) is the prototype species of the Parapoxvirus genus, which causes contagious ecthyma in sheep and goats. The disease is also known as Orf, contagious pustular dermatitis, infectious labial dermatitis, scabby mouth, and sore mouth. The disease, which is distributed worldwide and endemic in most sheep and/or goat-raising countries, is characterized by proliferative and self-limiting lesions around the muzzle and lips (scabby mouth) of infected animals, and sometimes also affects the gums and tongue, especially in young lambs [1,2]. The disease has a very high morbidity. Though mortality is low and usually does not exceed ten percent, mortality rates of up to 10% and 93% have been reported in lambs and kids [3-5]. The disease is frequently severe enough to create substantial welfare problems in flocks [6]. This, in turn, has an economic impact on sheep farmers due to the accompanying decreases in production.

In recent years, reports of severe Orf outbreaks in flocks have been gradually increased [7-10]. In addition, a mild form of the disease has been described in wild ruminants and in humans, in which is characterized by self-limiting, painful pustular lesions on the hands and fingers [11,12]. Several ORFV attenuated live vaccines have been used worldwide since 1981 and form the main prophylactic measure against contagious ecthyma in sheep and goats [13]. However, Conventional ORFV attenuated live vaccines are less effective at preventing the disease at present. It mainly due to the available vaccines do not induce lasting immunity in sheep and the rapid changes in the genomes of Orf virus vaccine strains during cell culture adaptation, particularly involving the ends of viral genome [14].

The host immune response to ORFV has been extensively studied, yet many aspects of the complex host-virus interactions remain unclear. Several studies have demonstrated that the major envelop proteins of ORFV could induce a strong immune response [15,16]. As a major immunogenic protein, the ORFV011 protein can induce a strong antibody response by stimulating lymphocytes derived from draining lymph nodes [17]. In addition, the potential of the ORFV059 protein to act as an antigen in subunit vaccines against antigenically identical Orf viral strains has been indicated. Furthermore, it seems to be responsible for induction of neutralizing antibodies in the host, and plays an important role in the viral cycle [15,18]. Considering the immunogenicity of the ORFV 011 and ORFV059 proteins, it is possible that the chimeric expression of the ORFV011 and ORFV059 proteins could induce stronger immune responses.

In this study, we assembled DNA vaccine plasmids expressing the two major immunodominant proteins (ORFV011 and ORFV059) of the Orf virus, individually and simultaneously. The expression of the recombinant proteins in vitro was investigated by western blotting analysis and indirect fluorescence antibody (IFA) tests. The levels of protective humoral and cellular immune responses induced by the recombinant ORFV DNA vaccines were investigated in a mouse model.

## Methods

### Virus and cells

A newly identified fatal strain of Orf virus was isolated from scab specimens collected from skin lesions of a 6-week-old small-tailed Han sheep affected with Orf virus in November 2008 in the Jilin province of China [9]. Primary ovine fetal turbinate (OFTu) cells were maintained in minimal essential medium (MEM) (Hyclone) supplemented with 10% fetal bovine serum (FBS) (Hyclone), 2 mM L-glutamine, 100 U of penicillin/ml, 100 μg of streptomycin/ml, and 20 μg of nystatin/ml. The virus was propagated in OFTu cells. For virus harvest, cell culture supernatant from infected cells were collected when approximately 90% of the culture showed cytopathic effects (CPE). After three freeze-thaw cycles, the supernatant was then cleared at 500 × g for 10 min at 4°C and stored at -80°C. The virus was purified by sucrose gradient centrifugation. Infectivity titre was assayed by the plaque method in OFTu cell culture and calculated as plaque forming units (PFU).

### Construction of the expression plasmids

All expression plasmids were constructed using pcDNA3.1(+) (Invitrogen, USA) as the vector. The two primer sets used for PCR amplification are specific for either ORFV 011 (forward primer: 5'-TATA**GGATCC**GCCATGT GGCCGTTCTCCTCCATC-3'; reverse primer: 5'-CCG**CTCGAG**TTAATTTATTGGCTTGCAG-3') (restriction sites in bold) or ORFV059 (forward primer: 5'-C**AAGCTT**GCCACCATGGATCCACCCGAAATC-3'; reverse primer: 5'-C**GAATTC**TCACACGATGGCCGTGACC-3') (restriction sites in bold). The plasmids pMDT-ORFV011 and pMDT-ORFV059, containing the complete ORFV011 and ORFV059 genes of the ORFV Jilin province strain, respectively, have been described previously [9]. The DNA vaccine constructs pcDNA3.1-ORFV011 and pcDNA3.1-ORFV059 expressing ORFV011 protein and ORFV059 protein individually were generated by subcloning the ORFV011 and ORFV059 genes into pcDNA3.1 (+), respectively. To construct the ORFV011/ORFV059 chimeric expression plasmid, the two primer sets mentioned above were modified by inserting a linker structure which encoded a (G_4_S)_2 _polypeptide in the 5' termini of the ORFV011 reverse primer and the ORFV059 forward primer. In addition, the stop codon of the ORFV011 reverse primer and the promotor of the ORFV059 forward primer were deleted. The modified primer sequences were as follows: ORFV011 (forward primer: 5'-TATA**GGATCC**GCCATGTGGCCGTTCTCCTCCATC-3'; reverse primer:5'-*AGAGCCTCCGCCACCGGATCCACCGCCACC*ATTTATTGGCTTGCAGAA-3') (linker structure in italic) and ORFV059 (forward primer: 5'- *GGTGGCGGTGGATCCGGTGGCGGAGGCTCT*GAAATCACGGGCTACATAATC-3'; reverse primer: 5'-C**GAATTC**TCACACGATGGCCGTGACC-3') (linker structure in italic). The coding sequence of the ORFV011 gene without the stop codon was amplified from pMDT-ORFV011 by PCR, using ORFV011 modified primers, and the gene for ORFV059 without the promoter was amplified from pMDT-ORFV059 using ORFV059 modified primers. PCR products, including the ORFV011 and ORFV059 genes obtained by overlap PCR, were cloned into a pcDNA3.1 (+) vector, generating pcDNA3.1-ORFV 011/ORFV059 plasmid. All recombinant plasmids (data not shown) were confirmed by restriction digestion and sequence analysis.

### Expression of recombinant proteins in vitro

OFTu cells were cultured in MEM containing 10% fetal bovine serum (FBS) in a 5% CO_2 _incubator at 37°C. Then, OFTu cells were transfected with pcDNA3.1(+) plasmid (control) or pcDNA3.1-ORFV011, pcDNA3.1-ORFV059 and pcDNA3.1-ORFV 011/ORFV059 recombinant vaccine plasmids. Briefly, OFTu cells were grown to 80% confluence in 6-well culture plates (Costar, Corning Incorporated, USA) and transfected with 8-10 μg of recombinant plasmids for each well, respectively. After 48 h post-transfection, cell lysates collected from pcDNA3.1(+) vector plasmid-transfected or pcDNA3.1-ORFV011, pcDNA3.1-ORFV059 and pcDNA3.1-ORFV059/ORFV011 recombinant vaccine plasmids-transfected OFTu cells were analyzed by SDS-PAGE and Western blot, respectively.

To further study the subcellular localization of target protein, the pcDNA3.1(+) vector plasmid-transfected or pcDNA3.1-ORFV011, pcDNA3.1-ORFV059 and pcDNA3.1-ORFV011/ORFV059 recombinant vaccine plasmids-transfected OFTu cells were subjected to an IFA test employing rabbit anti-ORFV polyclonal antibody (kindly provided by the Jilin Institute for Veterinary Research, diluted at 1:100). The secondary antibody used was FITC-conjugated goat anti-rabbit IgG (H + L) (1:500 dilution in PBS). After immunostaining, the normal control and transfected cells were observed under a fluorescent microscope.

### Immunization

Large-scale preparations of DNA vaccine plasmids were obtained using alkaline lysis. Sixty 6-8-week-old female Balb/c mice (purchased from the Laboratory Animal Center of Jilin University in China) were used for experiments in accordance with the guidelines for animal experimentation of Jilin University. The animals were maintained under pathogen-free conditions, randomly divided into six groups (ten mice per group), and immunized by skin scarification [19] with one of the following formulations: (π) the A group was immunized with PBS, (θ) the B group with 100 μg pcDNA3.1(+) plasmids, (ρ) the C group with 100 μg pcDNA3.1-ORFV011 plasmids, (σ) the D group with 100 μg pcDNA3.1-ORFV059 plasmids, (τ) the E group with 50 μg pcDNA3.1-ORFV011 plasmids plus 50 μg pcDNA3.1-ORFV059 plasmids, and (φ) the F group with 100 μg pcDNA3.1-ORFV011/ORFV059 plasmids. The mice were boosted in the same manner on days 14 and 28. Blood samples were collected from the tail artery at weeks 0, 1, 2, 3, 4, 5, 6, 7 and 8 post immunization. The sera were inactivated at 56°C for 30 min and stored at -80°C until assayed by ELISA (IgG at each time-points, IgG1/IgG2a isotype at 1 week post the final boost immunization, virus-neutralizing antibody at 2 weeks post immunization). On day 10 after the final boost immunization, 5 mice of each group were sacrificed by cervical dislocation, and their splenocytes were isolated for a lymphocyte proliferation assay and flow cytometry assay.

### ORFV-specific antibody titer assay

ORFV-specific antibody responses were determined using an indirect ELISA, with the purified ORFV as the antigen. Briefly, Costar High Binding 96-well plates (eBioscience, San Diego, CA) were coated overnight at 4°C with 0.1 μg of purified ORFV diluted in 100 μl of 0.1 M NaHCO_3 _buffer (pH 9.6). The plates were blocked with PBS/0.1% Tween-20/2% BSA (150 μl/well) for 1 h at 37°C. After washing three times with PBS/0.1% Tween-20, sera were added in a dilution of 1:20 in PBS/0.1% Tween-20/2% BSA. The plates were incubated for 90 min at 37°C, washed three times, and then incubated with 100 μl of horseradish-peroxidase (HRP)-conjugated goat anti-mouse IgG (Zhongshan Biotechnology Company, Beijing, China) at a dilution of 1:5000 in PBS/0.1% Tween-20/2% BSA for 1 h at 37°C. After three washes, the presence of IgG was detected with 100 μl of TMB according to the manufacturer's instructions. The reaction was stopped by adding 50 μl of 2 M H_2_SO_4_. The OD value was read at 450 nm. Results were expressed as an antibody endpoint titer, determined when the OD value is 3-fold greater than the background value obtained with a 1:20 dilution of serum from PBS-injected mice. The purified ORFV antigen was replaced with OFTu cell supernanant as a negative control.

### Immunoglobulin isotyping ELISA

In order to determine the IgG1 and IgG2a subtypes in mice immunized with ORFV vaccine plasmids, purified ORFV was diluted to optimal concentrations in 0.1 M NaHCO3 buffer (pH 9.6), which was used as coating antigen. Costar High Binding 96-well plates (eBioscience, San Diego, CA) were coated with 0.1 μg of purified ORFV diluted in 100 μl of 0.1 M NaHCO3 buffer (pH 9.6). The plates were blocked with PBS/0.1% Tween-20/2% BSA (150 μl/well) for 1 h at 37°C. After washing three times with PBS/0.1% Tween-20, the sera were diluted at 1:20 in PBS/0.1% Tween-20. After a 1 h incubation, the plates were washed with PBS/0.1% Tween-20 and goat anti mouse IgG1, IgG2a (Sigma Chemical Co., SA Locus, Mo.) diluted to 1:1000 were used to detect IgG1 and IgG2a subtypes, respectively. After incubating for 60 min, the plates were washed with PBS/0.1% Tween-20, and horseradish-peroxidase (HRP)-conjugated rabbit anti-goat IgG (Zhongshan Biotechnology Company, Beijing, China) diluted to 1:4000 in PBS/0.1% Tween-20 was added to each well. The reaction was visualized after 1 h of incubation with TMB. The reaction was stopped by adding 50 μl of 2 M H_2_SO_4_. The OD value was read at 450 nm. Results were expressed as an antibody endpoint titer, determined when the OD value is 3-fold greater than the background value obtained with a 1:20 dilution of serum from PBS-injected mice.

### Determination of virus-neutralizing titers

To perform the virus neutralization assay, serum samples were diluted 1:5 in MEM medium and heat-activated for 30 min at 56°C. This 1:5 starting dilution of inactivated sera was serially diluted two-fold in MEM medium in 96-well flat bottom plates and incubated with 0.1 μg of purified ORFV diluted in 50 μl MEM for 90 min at 37°C. All sera were run in triplicate. Subsequently, 100 μl of OFTu cell suspension were added to each well at a concentration of 45,000 cells/well. The plates were incubated for 5 days at 37°C in a 5% CO_2 _incubator. The neutralization titer of a serum is the dilution at which 50% of the wells are protected against virus infection. The titer is expressed as ND50 and calculated using the Reed and Muench method.

### Splenocyte preparations

Single-cell suspensions of spleens were prepared from the immunized mice at day 10 post the final boost immunization. Briefly, spleens from freshly killed mice were disrupted by using monofilament nylon filters, and the cells were collected and centrifuged at 500 × g for 20 min. The cell pellets were suspended in 10 ml RPMI 1640 and then centrifuged at 500 × g for 10 min at room temperature. The cells were resuspended in RPMI 1640 supplemented with 10% FBS at a concentration of 1 × 10^7 ^cells/ml before the lymphocyte proliferation assay and flow cytometry assay.

### Cellular proliferation assay

Untreated splenocytes were cultured in RPMI 1640 medium with 10% fetal serum. The cells were suspended in complete RPMI 1640 to achieve the concentration of 1 × 10^6 ^splenocytes/well. The primary incubation was at 37°C in a 5% CO_2 _incubator for 72 h, followed by addition of 10 μl MTT (5 mg/ml) per well and a secondary incubation at 37°C in a 5% CO_2 _incubator for another 4 h. The microplates were centrifuged, the unreacted MTT was removed, then 100 μl dimethyl sulfoxide (DMSO) was added per well to solubilize formazan. The plates were then incubated for 10 min with shaking. The absorbance of the plate was read using a 570 nm filter within minutes following shaking. The treated cells were cultured under the same conditions, but with the addition of purified ORFV antigen (at a final concentration of 5 μg/ml) in complete RPMI 1640 medium. The mitogen, Con A was added at 5 μg/ml concentration in the positive control wells. The geometric means and standard deviations for triplicate sets of samples were calculated. Lymphocyte proliferation is expressed as a stimulation index (SI), which is defined as the mean of experimental data divided by the mean of the unstimulated control [20,21].

### Flow cytometry assay

In order to determine the changes of specific T cell subsets in mice immunized with ORFV vaccine plasmids, the flow cytometry assay were performed. 1 × 10^6 ^splenocytes collected from the immunized mice at day 10 post the final boost immunization were suspended in PBS and stained with FITC-conjugated anti-mouse CD3, PE-Cy5-conjugated anti-mouse CD8, and PE-conjugated anti-mouse CD4 for 30 min on ice. After three washes with PBS, the cells were centrifuged at 500 × g for 10 min. The cells were suspended in 200 μl PBS. At least 10,000 cells were analyzed per sample. The stained cells were analyzed by a BD FACSAria flow cytometer, and the data were analyzed with Cellquest V3.3 software.

### ELISA assay

The production of ORFV-specific cytokines (IL-2, IL-4, IL-6, IFN-γ and TNF-α) was assessed by culturing splenocytes (1 × 10^6 ^cells/ml) in triplicate with purified ORFV antigen (5 μg/ml). Control stimuli included RPMI 1640 medium alone or ConA at 5 μg/ml. The supernatants were harvested after 72 h at 37°C in a 5% CO_2 _incubator, filtered and stored at -20°C until assayed. The presence of IL-2, IL-4, IL-6, IFN-γ and TNF-α in mouse splenocytes culture supernatants were determined by commercial cytokines immunoassay kits (Dakewa Biotech Company, Shenzhen, China) according to the manufacturer's instructions. Supplied standards were used to generate a standard curve. The detection limits of the assay were 2.5 pg/ml, 3.0 pg/ml, 3.5 pg/ml, 2.0 pg/ml, 2.0 pg/ml for IL-2, IL-4, IL-6, IFN-γ and TNF-α, respectively.

### Statistical analysis

All data were analyzed using the statistical software program Systat 10 (SPSS). The distribution of data was determined using descriptive statistics. Data which were not normally distributed were transformed by ranking. Differences in ELISA titers were investigated using one-way analysis of variance (ANOVA) performed on the rank. The means of the rank-transformed variables were compared using Tukey's multiple comparison test. Values of * *P *< 0.05, ** *P *< 0.01 were considered statistically significant.

## Results

### Expression of ORFV011 protein, ORFV059 protein and ORFV011/ORFV059 chemeric-proteins *in vitro*

**Figure 1:**
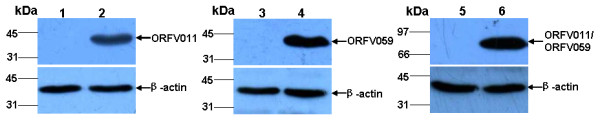
**Western blot analysis of ORFV011 protein, ORFV059 protein and ORFV 011/ORFV 059 chemeric-proteins**. OFTu cells were transfected with pcDNA 3.1(+) plasmid (control) or pcDNA 3.1-ORFV 011, pcDNA 3.1-ORFV 059, pcDNA 3.1-ORFV 011/ORFV 059 vaccine plasmids, respectively. After 48 h post-transfection, lysates collected from different vaccine plasmids-transfected OFTu cell were examined by SDS-PAGE -Western blotting with rabbit anti-ORFV polyclonal antibody (kindly provided by the Jilin Institute for Veterinary Research, diluted at 1:100). ORFV011 protein, ORFV059 protein and ORFV 011/ORFV059 chimeric-proteins bands could be detected in pcDNA3.1-ORFV011, pcDNA3.1-ORFV059 and pcDNA3.1-ORFV011/ORFV059 recombinant vaccine plasmids-transfected OFTu cell lysates (Figure 1, Lanes 2, 4, 6), but no corresponding protein bands were detected in the pcDNA3.1(+)-transfected cell lysates (Figure 1, Lanes 1, 3, 5). Lanes 2, 4, 6: OFTu cells transfected with pcDNA 3.1-ORFV011, pcDNA 3.1-ORFV059 or pcDNA 3.1-ORFV011/ORFV059 vaccine plasmids, respectively. Lanes 1, 3, 5: OFTu cells transfected with pcDNA 3.1(+) plasmid (control).

**Figure 2:**
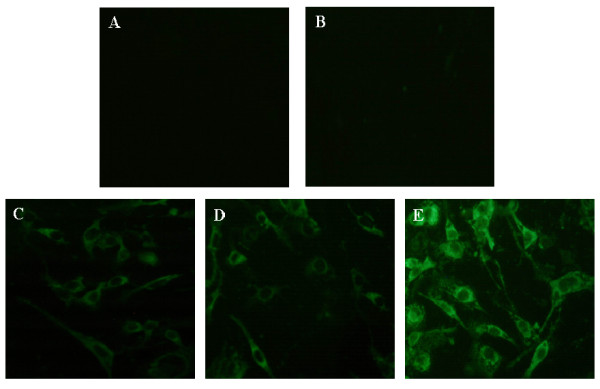
**The intracellular location of ORFV011 protein, ORFV059 protein and ORFV 011/ORFV059 chemeric-proteins in transfected OFTu cells**. The OFTu cells were seeded in 6-well culture plates and un-transfected (Figure 2a) or transfected with pcDNA3.1(+) vector (Figure 2b), pcDNA3.1-ORFV011 plasmid (Figure 2c), pcDNA3.1-ORFV059 plasmid (Figure 2d) or pcDNA3.1-ORFV011/ORFV059 plasmid (Figure 2e) for 48 h. After 48 h post-transfection, the cells were fixed with 80% acetone for 10 min at -20°C, rehydrated in PBS, labeled with a rabbit anti-ORFV polyclonal antibody, and washed three times with PBS. FITC-conjugated goat anti-rabbit IgG (H + L) (1:500 dilution in PBS) was added to the OFTu cell mixtures for 30 min at room temperature, and the cells were washed and observed with a fluorescence microscopy. Magnification: 400 ×.

To confirm whether the pcDNA3.1-ORFV011, pcDNA3.1-ORFV059 and pcDNA3.1-ORFV011/ORFV059 vaccine plasmids expressing ORFV011 protein, ORFV 059 protein and ORFV011/ORFV059 chimeric-proteins in vitro, the pcDNA3.1-ORFV 011, pcDNA3.1-ORFV059 and pcDNA3.1-ORFV011/ORFV059 recombinant vaccine plasmids-transfected OFTu cell lysates were analyzed by SDS-PAGE and Western blot, respectively. A pcDNA3.1(+) vector was used as negative control. Western blot analysis revealed that ORFV011 protein, ORFV059 protein and ORFV011/ORFV059 chimeric- proteins bands could be detected in pcDNA3.1-ORFV011, pcDNA3.1-ORFV059 and pcDNA3.1-ORFV011/ORFV059 recombinant vaccine plasmids-transfected OFTu cell lysates (Figure 1, Lanes 2, 4, 6), but no corresponding protein bands were detected in the pcDNA3.1(+)-transfected cell lysates (Figure 1, Lanes 1, 3, 5). Beta-actin was probed as an internal control. In addition, the IFA tests on the pcDNA3.1-ORFV011, pcDNA3.1-ORFV059 and pcDNA3.1-ORFV011/ORFV059 vaccine plasmids-transfected cells were performed in order to further identify the expression of ORFV011 protein, ORFV059 protein and ORFV011/ORFV059 chimeric-proteins and study their subcellular localization. After 48 h post-transfection, the specific green fluorescence signal could be detected in the cytoplasm of pcDNA3.1-ORFV011, pcDNA3.1-ORFV059 and pcDNA3.1-ORFV011/ORFV059-transfected cells, repectively (Figure 2c, d and 2e), but no fluorescence was found in untransfected cells or pcDNA3.1(+)-transfected cell (Figure 2a and 2b).

### Specific antibody immune responses of mice to ORFV in different immunized groups

**Figure 3:**
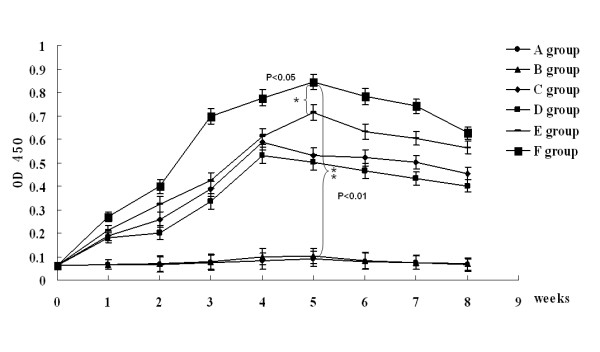
**Group mean optical density ratios and standard errors for specific anti-ORFV-IgG antibody responses in the serum samples of immunized mice of different groups**. The sera were collected from mice in groups (pcDNA3.1-ORFV011 group, pcDNA3.1-ORFV059 group, pcDNA3.1-ORFV011/ORFV059 group, pcDNA3.1-ORFV 011 plus pcDNA3.1-ORFV059 group, pcDNA3.1(+) group and PBS group) at weeks 0, 1, 2, 3, 4, 5, 6, 7, 8 and were inactivated at 56° for 30 min. The ORFV-specific antibody responses were determined using an indirect ELISA, with the purified ORFV as the coating antigen. As shown in Figure 3, the levels of anti-ORFV-IgG antibodies were developed in different DNA vaccine plasmid- immunized group. One week after the final boost immunization, the level of anti-ORFV-IgG antibodies was higher in the pcDNA3.1-ORFV 011/ORFV 059 immunized group than any of the other groups tested (OD_450 _value: 0.829 ± 0.014, **P < 0.01). The levels of anti-ORFV-IgG antibodies in both of pcDNA3.1-ORFV 011 group and pcDNA3.1-ORFV 059 group were very nearly, but in the pcDNA3.1-ORFV 011 group increased slightly compared with the pcDNA3.1-ORFV 059 group (OD_450 _value: 0.579 ± 0.009 vs 0.518 ± 0.003, *P *> 0.05). The levels of anti-ORFV-IgG antibodies was higher in the pcDNA3.1-ORFV 011/ORFV 059 group than pcDNA3.1-ORFV 011 plus pcDNA3.1-ORFV 059 group (OD_450 _value: 0.829 ± 0.014 vs 0.687 ± 0.024, *P < 0.05). A group: PBS; B group: pcDNA3.1(+) vector; C group: pcDNA3.1-ORFV011; D group: pcDNA3.1-ORFV059; E group: pcDNA3.1-ORFV011 plus pcDNA3.1-ORFV059; F group: pcDNA3.1-ORFV011/ORFV059.

**Figure 4:**
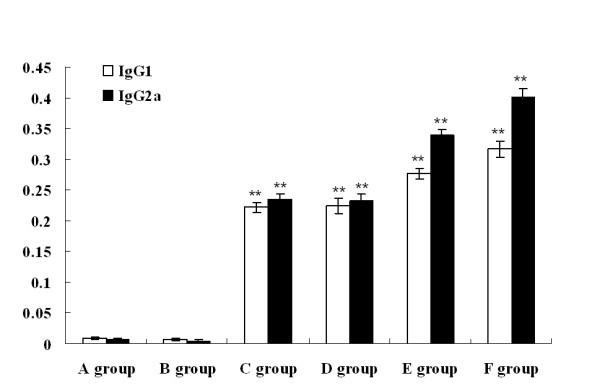
**The subclasses IgG1 and IgG2a of anti-ORFV IgG antibodies in the serum samples of immunized mice of different groups**. Sera were taken from immunized mice of different groups 1 week post the final boost immunization. The titration of ORFV-specific immunoglobulin (IgG) subclasses 1 and 2a was detected by ELISA. As shown in Figure 4, the assay of IgG2a and IgG1 antibodies performed at 1 week post the final boost immunization indicates a significant difference between vaccine plasmids groups (including pcDNA 3.1-ORFV011 group, pcDNA 3.1-ORFV059 group, pcDNA3.1-ORFV011 plus pcDNA3.1-ORFV059 group and pcDNA3.1-ORFV011/ORFV059 group) and control groups (including PBS and pcDNA3.1(+) group) (**P < 0.01). A group: PBS; B group: pcDNA3.1(+) vector; C group: pcDNA3.1-ORFV011; D group: pcDNA3.1-ORFV059; E group: pcDNA3.1-ORFV011 plus pcDNA3.1-ORFV059; F group: pcDNA3.1-ORFV011/ORFV059.

**Figure 5:**
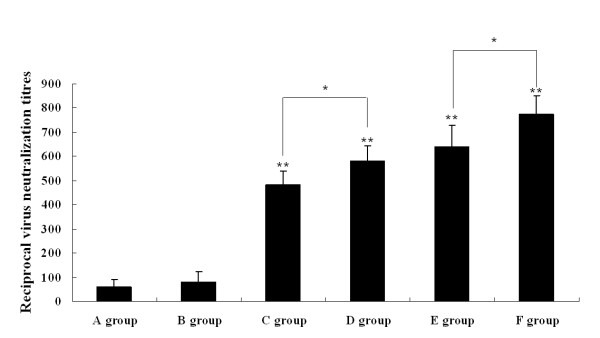
**Virus-neutralizing titer assay**. The virus-neutralizing titers were determined in sera pooled from immunized mice of different groups 2 weeks post immunization. The sera (diluted 1:5 in MEM medium) were heat-activated for 30 min at 56°C, then serially diluted two-fold in 96-well flat bottom plates and incubated with 0.1 μg of purified ORFV diluted in 50 μl MEM for 90 min at 37°C. All sera were run in triplicate. Subsequently, 100 μl of OFTu cell suspension were added to each well at a concentration of 45,000 cells/well. The plates were incubated for 5 days at 37°C in a 5% CO_2 _incubator. The titer is expressed as ND50 and calculated using the Reed and Muench method. As shown in Figure 5, the assay of virus-neutralizing titers performed at 2 weeks post immunization indicates a significant difference between four vaccine plasmids groups (including pcDNA 3.1-ORFV 011 group, pcDNA 3.1-ORFV 059 group, pcDNA-ORFV 011 plus pcDNA-ORFV 059 group and pcDNA-ORFV 011/ORFV 059 group) and control groups (including PBS and pcDNA3.1(+) group) (**P < 0.01). Among the four vaccine plasmids groups, the virus-neutralizing titers in the pcDNA-ORFV 011/ORFV 059 group was significantly higher than that of pcDNA-ORFV 011 plus pcDNA-ORFV 059 group (*P < 0.05). A group: PBS; B group: pcDNA3.1(+) vector; C group: pcDNA3.1-ORFV011; D group: pcDNA3.1-ORFV059; E group: pcDNA3.1-ORFV011 plus pcDNA3.1-ORFV059; F group: pcDNA3.1-ORFV011/ORFV059.

To determine the immunogenicity of pcDNA3.1-ORFV011, pcDNA3.1-ORFV059, pcDNA3.1-ORFV011/ORFV059 and pcDNA3.1-ORFV011 plus pcDNA3.1-ORFV059 vaccine plasmids, four groups of the mice were respectively immunized by skin scarification with vaccine plasmids mentioned above. Two additional groups received either pcDNA3.1(+) vector plasmids or PBS. To investigate the humoral immune responses in mice induced by different DNA vaccine plasmids, sera from mice in groups (pcDNA3.1-ORFV011 group, pcDNA3.1-ORFV059 group, pcDNA3.1-ORFV 011/ORFV059 group, pcDNA3.1-ORFV011 plus pcDNA3.1-ORFV059 group, pcDNA3.1(+) group and PBS group) were collected weekly and anti-ORFV-IgG antibodies in these sera were detected by ELISA. As shown in Figure 3, the levels of anti-ORFV-IgG antibodies were developed in different DNA vaccine plasmid- immunized group. One week after the final boost immunization, the level of anti-ORFV-IgG antibodies was higher in the pcDNA3.1-ORFV011/ORFV059 immunized group than any of the other groups tested (OD_450 _value: 0.829 ± 0.014, **P < 0.01). The levels of anti-ORFV-IgG antibodies in both of pcDNA3.1-ORFV011 group and pcDNA3.1-ORFV059 group were very nearly, but in the pcDNA3.1-ORFV011 group increased slightly compared with the pcDNA3.1-ORFV059 group (OD_450 _value: 0.579 ± 0.009 vs 0.518 ± 0.003, P > 0.05). The levels of anti-ORFV-IgG antibodies was higher in the pcDNA3.1-ORFV011/ORFV059 group than pcDNA3.1-ORFV011 plus pcDNA3.1-ORFV059 group (OD_450 _value: 0.829 ± 0.014 vs 0.687 ± 0.024, *P < 0.05). In addition, ORFV-specific immunoglobulin G (IgG) subclasses 1 and 2a were also detected 1 week after the final immunization (Figure 4). In both pcDNA3.1 (+) plasmids and PBS groups, the levels of IgG1 and IgG2a were fairly low. However, the levels of anti-ORFV IgG1 and IgG2a in DNA vaccine plasmids immunizations groups were higher than control groups (including pcDNA3.1 (+) group and PBS group). We further examined whether there was any difference in the type of T helper responses among these groups by using the IgG2a/IgG1 ratio as a surrogate marker. Interestingly, the ratios of IgG2a/IgG1 in mice receiving DNA vaccine plasmids immunizations were more than 1, indicating that DNA vaccine plasmid constructs induced predominantly a Th1-type immune response after the final boost immunization. In addition, the virus-neutralizing titers were determined in sera pooled from immunized mice of different groups 2 weeks post immunization. As shown in Figure 5, the neutralizing activity against ORFV was detected at a high level in sera of mice immunized with DNA vaccine plasmid constructs. The assay of virus-neutralizing titers indicated a significant difference between four vaccine plasmids groups (including pcDNA3.1-ORFV011 group, pcDNA3.1-ORFV059 group, pcDNA3.1-ORFV011 plus pcDNA3.1-ORFV059 group and pcDNA3.1-ORFV011/ORFV059 group) and control groups (including PBS and pcDNA3.1(+) group) (**P < 0.01). Among the four vaccine plasmids groups, the virus-neutralizing titer in the pcDNA3.1-ORFV011/ORFV059 group was significantly higher than that of pcDNA3.1-ORFV011 plus pcDNA3.1-ORFV059 group (*P < 0.05).

### Proliferation-inducing effect of splenocytes in different immunized groups

**Figure 6:**
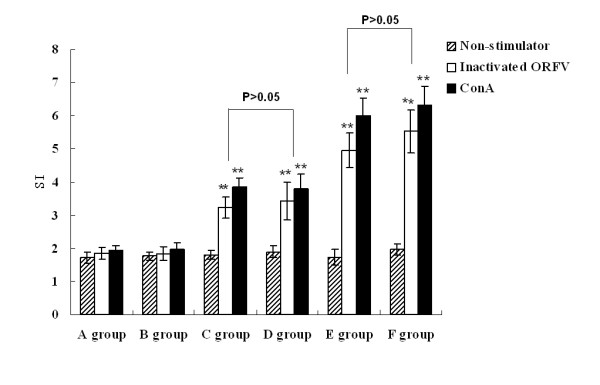
**Proliferation of spleen lymphocytes in the different groups**. Six groups of 6-8-week-old female Balb/c mice were immunized by skin scarification with one of the following formulations: (A) PBS; (B) pcDNA3.1(+) vector; (C) pcDNA3.1-ORFV011; (D) pcDNA3.1-ORFV059; (E) pcDNA3.1-ORFV011 plus pcDNA3.1-ORFV059; (F) pcDNA3.1-ORFV011/ORFV059. Each bar represents the group mean (*N *= 10) ± S.E.M. of SI determined in triplicate. The mitogen, Con A served as a positive antigen and positive control. As shown in Figure 5, mice immunized with pcDNA3.1-ORFV011/ORFV059 (F group) induced a stronger proliferation response compared with background OD (0.281 ± 0.123) (**P < 0.01). The E group, immunized with pcDNA3.1-ORFV011 plus pcDNA3.1-ORFV059, also resulted displayed a higher stimulation index (SI) than mice immunized with PBS, pcDNA3.1 (+) or vaccine plasmid alone. However, the stimulation index (SI) in pcDNA3.1-ORFV011/ORFV059 group increased slightly compared with the pcDNA3.1-ORFV011 plus pcDNA3.1-ORFV059 group (*P *> 0.05), and the stimulation index (SI) were no obvious difference between pcDNA3.1-ORFV 011 group and pcDNA3.1-ORFV 059 group (*P *> 0.05).

The ability of ORFV DNA vaccine plasmids to mediate spleen lymphocyte proliferation was measured by a MTT colorimetric method. Representative results of a proliferation study using prepared splenocytes of various groups are shown in Figure 6. Compared to background OD (SI = 0.281 ± 0.123), mice immunized with pcDNA3.1-ORFV 011/ORFV 059 (F group) induced a stronger proliferation response (**P < 0.01). The E group, immunized with pcDNA3.1-ORFV 011 plus pcDNA3.1-ORFV 059, also resulted displayed a higher stimulation index (SI) than mice immunized with PBS, pcDNA3.1 (+) and vaccine plasmid alone (pcDNA 3.1-ORFV011 or pcDNA 3.1-ORFV059). However, the stimulation index (SI) in pcDNA3.1-ORFV011/ORFV059 group increased slightly compared with the pcDNA3.1-ORFV011 plus pcDNA3.1-ORFV 059 group (*P *> 0.05), and the stimulation index (SI) were no obvious difference between pcDNA3.1-ORFV011 group and pcDNA3.1-ORFV059 group (*P *> 0.05).

### Subsets of specific T lymphocyte cells

**Figure 7:**
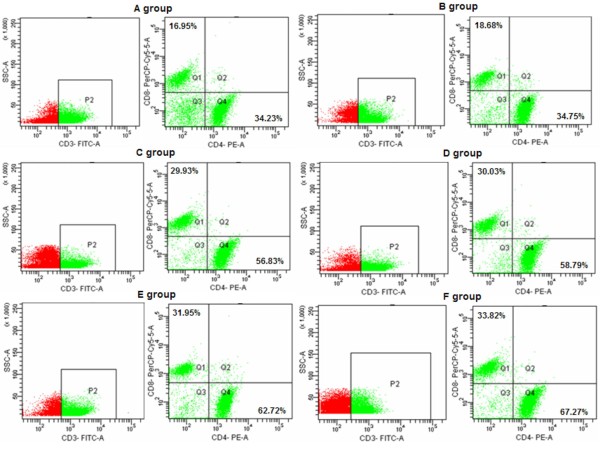
**The CD4^+ ^and CD8^+ ^T lymphocyte subsets in the different groups were assessed by FACS analysis at day 10 post the final boost immunization**. Spleen lymphocytes of mice in different immunized groups were repectively enriched from the spleens at days 10 post the final boost immunization and cultured in RPMI 1640 medium. The CD4^+ ^and CD8^+ ^T lymphocyte subsets in the different groups were assessed by FACS in order to examine whether or not DNA vaccine constructs immunized mice by skin scarification can induce a T-cell response in mice and further activate cell immunity response. (A) PBS; (B) pcDNA3.1(+) vector; (C) pcDNA3.1-ORFV011; (D) pcDNA3.1-ORFV059; (E) pcDNA3.1-ORFV011 plus pcDNA3.1-ORFV059; (F) pcDNA3.1-ORFV011/ORFV059.

To examine whether or not immunized mice by skin scarification developed an induced T-cell response against ORFV, T lymphocyte cells from mice in groups (pcDNA3.1-ORFV011 group, pcDNA3.1-ORFV059 group, pcDNA3.1-ORFV 011/ORFV059 group, pcDNA3.1-ORFV 011 plus pcDNA3.1-ORFV group, pcDNA3.1(+) group and PBS group) were respectively enriched from the spleens 10 days after the final boost immunization and cultured in RPMI 1640 medium. As shown in Figure 7, the percentages of CD4^+ ^and CD8^+ ^cells in mice immunized with DNA vaccine plasmids increased significantly compared to mice immunized with the PBS and pcDNA3.1(+). The ratios of CD4^+^/CD8^+ ^cells in mice immunized with vaccine plasmids were higher than the control groups. These results further indicate that the DNA vaccine constructs can induce a T-cell response in mice and activate cell immunity response.

### ORFV-specific cytokine production in the splenocytes culture supernatants

**Figure 8:**
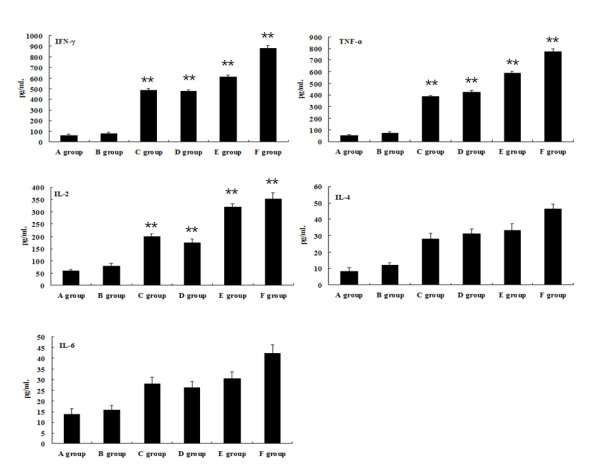
**The production of ORFV-specific cytokines in the supernatant of splenocytes stimulated with purified ORFV at day 10 post the final boost immunization**. Six groups of of 6-8-week-old female Balb/c mice were immunized by skin scarification with one of the following formulations: (A) PBS; (B) pcDNA3.1(+) vector; (C) pcDNA3.1-ORFV011; (D) pcDNA3.1-ORFV059; (E) pcDNA3.1-ORFV011 plus pcDNA3.1-ORFV059; (F) pcDNA3.1-ORFV011/ORFV059. The levels of cytokines production (IFN-γ, TNF-α, IL-2, IL-4 and IL-6) in the supernatant of splenocytes stimulated with purified ORFV were determined by ELISA assay. Each bar represents the group mean (*N *= 10) ± S.E.M. of cytokines levels determined in triplicate.

We also investigated the responsiveness of spleen cells from immunized mice against ORFV. We determined the levels of cytokines production (IFN-γ, TNF-α, IL-2, IL-4 and IL-6) in the supernatant of splenocytes stimulated with purified ORFV. Little levels of IFN-γ, TNF-α and IL-2 were observed in mice immunized with empty vector or PBS. The levels of IFN-γ, TNF-α and IL-2 produced by splenocytes immunized with ORFV DNA vaccine plasmids were significantly higher than that with pcDNA3.1 (+) or PBS (**P < 0.01). The levels of Th2 type cytokines (IL-4 and IL-6) increased slightly in ORFV DNA vaccine plasmids immunized mice compared with pcDNA3.1 (+) or PBS immunized mice, but the changes were not so dramatic(Figure 8). The results mentioned above indicated that an efficient antigen-specific T cell activation, most probably CD8^+ ^T cells, was induced by ORFV DNA vaccine plasmids expressing the MHC class I epitope.

## Discussion

The continuing occurrence of Orf in the ovine and caprine herds all over the world emphasizes the need to develop novel vaccines with improved properties compared to the currently used attenuated live vaccines. One promising approach is the use of viral DNA vaccines because they are safe and can induce both neutralizing antibodies and cellular immune responses while maintaining the advantages of subunit and traditional vaccines [22,23]. To our knowledge, the current study is the first report demonstrating the potential of an ORFV DNA vaccine to induce strong immune responses in mice.

ORFV 011 and ORFV 059 are two major immunodominant proteins which have been demonstrated to induce a strong humoral response against ORFV [17,24]. In our study, ORFV DNA vaccines can induced specific humoral and cellular responses in mice. The levels of ORFV-specific IgG antibodies were greatly enhanced following two boosts. In addition, the higher neutralizing antibodies were detected in pcDNA3.1-ORFV 059, pcDNA3.1-ORFV 011 plus pcDNA3.1-ORFV 059 or pcDNA3.1-ORFV 011/ORFV 059 groups. Our findings are supported by a previous study showing that ORFV 059 protein could induce the production of neutralizing antibodies [15]. The measurement of certain antibody isotypes correlates directly to type 1 or type 2 T cell responses [25]. For example, IgG2a in mice [26] is associated with in vivo production of IFN-γ and can be used as an indicator to measure the type 1 responses. Here, the IgG2a:IgG1 isotypes in immunized mice were assayed by ELISA. The levels of anti-ORFV IgG1 and IgG2a in DNA vaccine plasmids immunizations groups were higher than control groups. We further examined whether there was any difference in the type of T helper responses among these groups by using the IgG2a/IgG1 ratio as a surrogate marker. Interestingly, the ratios of IgG2a/IgG1 in mice receiving DNA vaccine plasmids immunizations were more than 1, indicating that DNA vaccine plasmid constructs induced predominantly a Th1-type immune response after the final boost immunization.

Next, T-cell immune responses were also observed in our study. T lymphocytes are generally divided into helper (CD4^+^) and CTL (CD8^+^) cells. CD4^+ ^T cells can increase the number of memory cells. The memory cells respond rapidly when re-exposed to pathogens and thus play a vital role in protection against viral challenge [27,28]. After activation, naive antigen-specific CD8^+ ^T cells are able to proliferate quickly and differentiate into potent effector cells capable of rapid cytokine production and cytolytic killing of target cells [29]. CD4^+ ^lymphocytes are further subdivided into Th1 and Th2 on the basis of the type of released cytokines. The Th1 cell subset mainly includes cells secreting IL-2, TNF-α, and INF-γ. The main roles of these cytokines include enhancing killer cell cytotoxicity and cell-mediated immune response. The Th2 cell subset mainly includes cells producing IL-4, IL-6, and IL-10. The role of Th2 cytokines is to promote antibody production and mediate humoral immune response. In the study, we investigated the responsiveness of spleen cells from immunized mice against ORFV. The levels of cytokines production (IFN-γ, TNF-α, IL-2, IL-4 and IL-6) in the supernatant of splenocytes stimulated with purified ORFV were determined by ELISA assay. The levels of IFN-γ, TNF-α and IL-2 produced by splenocytes immunized with ORFV DNA vaccine plasmids were significantly higher than that with pcDNA3.1 (+) or PBS (**P < 0.01). The result indicated that an efficient antigen-specific T cell activation, most probably CD8^+ ^T cells, was induced by ORFV DNA vaccine plasmids expressing the MHC class I epitope.

To further address the T cell response, we examined the CD4^+ ^and CD8^+ ^splenocytes collected from immunized mice by flow cytometry analysis. The percentages of CD4^+ ^and CD8^+ ^cells in mice immunized with DNA vaccine plasmids increased significantly compared to mice immunized with the PBS and pcDNA3.1(+). The ratios of CD4^+^/CD8^+ ^cells in mice immunized with vaccine plasmids were higher than the control groups. These results further indicate that the DNA vaccine constructs can induce a T-cell response in mice and activate cell immunity response.

Taken together, these data indicate that in vaccinated mice, the ORFV011 and ORFV059 can be expressed and secreted, induce specific humoral and cellular immune responses by activate both B and T cells, and the chimeric expression of ORFV011 and ORFV059 could significantly improve the potency of DNA vaccination. We concluded that DNA vaccine pcDNA3.1-ORFV011/ORFV059 expressing ORFV011 and ORFV059 chemeric-proteins can significantly improve the potency of DNA vaccination and could be served as more effective and safe approach for new vaccines against ORFV. Further studies are being carried out in lambs to understand the similarities and differences in the response between mice and lambs. These studies will provide a basis for the development of an ORFV DNA vaccine ready for clinical trial.

## Conclusions

We concluded that DNA vaccine pcDNA3.1-ORFV011/ORFV059 expressing ORFV011 and ORFV059 chemeric-proteins can significantly improve the potency of DNA vaccination and could be served as more effective and safe approach for new vaccines against ORFV.

## Abbreviations

ORFV: Orf virus; IFA: Indirect fluorescence antibody; OFTu: Primary ovine fetal turbinate; MEM: Minimal essential medium; FBS: Fetal bovine serum; CPE: Cytopathic effects; PFU: Plaque forming units; SI: Stimulation index.

## Competing interests

The authors declare that they have no competing interests.

## Authors' contributions

KZ, WH and WG carried out most of the experiments and wrote the manuscript. HL participated in the construction of expression plasmids. TH identified the expression of recombinant proteins in vitro. JL and XZ carried out the animal immunization experiment. BZ, GW and GS participated in the investigation of the levels of protective humoral and cellular immune responses induced by the recombinant ORFV DNA vaccines. ZZ participated in the design of the study. FG and DS conceived of the study and participated in its design and coordination. All authors read and approved the final manuscript.
